# A novel monoclonal antibody associated with glucoside kills gastric adenocarcinoma AGS cells based on glycosylation target

**DOI:** 10.1111/jcmm.17504

**Published:** 2022-08-09

**Authors:** Heng Xu, Boying Dun, Beiyi Liu, David Mysona, Jin‐Xiong She, Rong Ma

**Affiliations:** ^1^ Jiangsu Provincial Institute of Materia Medica Nanjing Tech University Nanjing China; ^2^ Jinfiniti Precision Medicine Augusta Georgia USA; ^3^ Institute of Animal Science Jiangsu Academy of Agricultural Science Nanjing Jiangsu China; ^4^ Research Center For Clinical Oncology Jiangsu Cancer Hospital and Jiangsu Institute of Cancer Research and Nanjing Medical University Affiliated Cancer Hospital Nanjing China

**Keywords:** cancer, cell death, glucoside, glycosylation, monoclonal antibody

## Abstract

Glycosylation results in the production of glycans which are required for certain proteins to function. These glycans are also present on cell surfaces where they help maintain cell membrane integrity and are a key component of immune recognition. As such, cancer has been shown to alter glycosylation to promote tumour proliferation, invasion, angiogenesis, and immune envasion. Currently, there are few therapeutic monoclonal antibodies (mAb) which target glycosylation alterations in cancer. Here, we report a novel mAb associated with a glucoside, mAb 201E4, which is able induce cancer cell death and apoptosis based on a specific glycosylation target. This mAb evokes cancer cell death in vitro via caspase, fas, and mitochondrial associated apoptotic pathways. The efficacy of this mAb was further confirmed in vivo as treatment of mice with mAb 201E4 resulted in potent tumour shrinkage. Finally, the antibody was proven to be specific to glycosylation alterations in cancer and have no binding to normal tissues. This data indicates that mAb 201E4 successfully targets glycosylation alterations in neoplasms to induce cancer cell death, which may provide a new strategy for therapy in cancer.

## 1 INTRODUCTION

Antibody based therapies for cancer have become widely adopted in the current era due to their ability to improve patient survival with few side effects. Examples include bevacizumab, cetuximab, rituximab, trastuzumab, and pembrolizumab.^1^, ^2^, ^3^ These monoclonal antibodies (mAbs) are unable to directly induce cell death. Instead, they exploit specific pathways, such as angiogenesis, or rely on immune effectors such antibody‐dependent cellular cytotoxicity (ADCC) or complement‐dependent cytotoxicity (CDC).^4^, ^5^ However, tumours may develop resistance to these mAbs via stimulating growth through unrelated pathways, altering cell surface receptors, or immune evasion.^6^ Thus, additional mAb options are needed which are tumour specific and able to directly stimulate cancer cell death without the need of additional pathways or effectors. Glycans, a class of molecules which alter lipids and proteins, may offer a new target that can accomplish these goals.

Abnormal glycan modifications are a hallmark of tumour cells that could be an ideal target for mAb targeting. These molecules have a vast array of functions, which tumours use to their advantage to stimulate proliferation, invasion, angiogenesis, and immune evasion.^7^, ^8^, ^9^, ^10^ Aberrant O‐glycosylation contributes to the development of colorectal cancer through direct induction of oncogenic properties in cancer cells.^11^ O‐GlcNAcylation promotes colorectal cancer progression by regulating protein stability.^12^ Furthermore, glycans have been shown to contribute to resistance to cytotoxic chemotherapy and mAbs, such as transtuzumab and pembrolizumab.^3^, ^13^ To date, there are few anti‐glycan associated mAbs secondary to that technology has only recently come available to aide in uncovering and isolating glycans, as well as design antibodies capable of recognizing them.^14^, ^15^, ^16^

We report the discovery of a novel immunoglobulin G3 (IgG3) mAb, named “mAb 201E4”, which is glycan specific. Antigen characterization showed that mAb 201E4 recognizes various glycans but does not bind to any specific protein. Surprisingly, mAb 201E4 directly evoked tumour cells death and its was confirmed in vivo with xenograft models. These results highlight that mAb 201E4 represents a unique mAb targeting glycans which can directly cause cancer cell death through the binding to various post translational glycosylation modifications.

## 2 MATERIAL AND METHODS

### 2.1 Cell lines, mouse, reagents, monoclonal antibody

Most cell lines were obtained from the American Type Culture Collection (ATCC) and cultured according to ATCC protocols. Human normal oesophageal epithelial cell (HEEC) was purchased from yuchi biology (Shanghai, China). Human normal ovarian epithelial cell (IOSE80) was purchased from guandao biology (Shanghai, China). MCF‐7 and Hs 746 T cells were grown in Dulbecco's modified Eagle's medium (DMEM) (GIBCO, USA), and all other cells were grown in RPMI 1640 (GIBCO, USA) at 37°C with 5% CO_2_. Both media were supplemented with 10% fetal bovine serum (FBS) (Hyclone, USA). The BALB/c nude mice (6 weeks old) were purchased from Frederick National Laboratory for Cancer Research (USA). PNGase F was purchased from New England Biolabs (USA). The novel monoclonal antibody (mAb) was developed, identified and named 201E4 (murine, IgG3 subtype) by Jinfiniti Precision Medicine. The mAb 201E4 was developed via hybridoma technology. The use of experimental animals is accordant to the Guidelines for the Management and Use of Laboratory Animals (USA). All of the procedures were approved by the animal care committee of the animal facility (Georgia Regents University, USA). The myeloma cells fused with the spleen cells harvested from female BALB/c mice (Frederick National Laboratory for Cancer Research, USA) which immunized with gastric adenocarcinoma AGS cells by standard immunization procedure. According to the research purpose, about 5000 positive clones were screened through a specific scheme till 201E4 was determined. The supernatants of hybridoma cells (mAb 201E4) were purified using Protein G (Invitrogen). The mAb 201E4 was stored at −80°C after purification.

### 2.2 Cytotoxicity assay

Cell viability was determined using cell counting kit‐8 (CCK‐8, Dojindo Laboratories). In brief, cancer cells were seeded into 96‐well culture plates at a density of 3 × 10^3^ (50 μl per well), then incubated for 24 h at 37°C. After incubation, according to the mAb concentration, the mAb was serially twofold diluted in medium from 200 μg/ml, respectively. Then, 50 μl of corresponding mAb were mixed with cells at an equal volume. After incubation for 72 h at 37°C, cells were incubated with 3 μl CCK‐8 for 4 h at 37°C. Then, the absorbance was measured at 450 nm by use of a microplate reader (Thermo). Wells with untreated cells or with human dermal fibroblasts cells were used as positive and negative controls, respectively. Growth inhibition curves were plotted as a percentage of untreated control cells, according to standard curves. The IC_50_ values of mAb 201E4 against cancer cells were calculated.

### 2.3 Tumour xenograft studies

2 × 10^6^ AGS or HCT‐8 cells in 100 μl PBS with matrigel (BD Biosciences, USA) (1:1)/mouse were inoculated subcutaneously in the right leg flank. For xenograft groups, mice were treated with mAb 201E4 immediately after cancer cell inoculation (prevention group) or at day 14 later when the implanted tumour had grown to 50–100 mm^3^ (therapeutic group). Groups of mice were treated with 201E4 mAb (25 mg/kg) and control mice were treated with the same concentrations of non‐specific mouse immunoglobulin G (IgG) (Invitrogen). Tumour size was measured by callipers per 3 days post‐treatment with the mAb and calculated as 0.5 × length × width.^2^ Tumours were weighed after sacrifice of the mice.

### 2.4 Cell cycle analysis and cell apoptosis analysis

AGS cells were cultured in two different concentrations of mAb 201E4 (100 and 200 μg/ml) for 24 h at 37°C under 5% CO_2_. The same concentrations of non‐specific mouse IgG (Invitrogen) were used as isotype controls. AGS cells were harvested, fixed with 70% ethanol at 4°C overnight, and then stained with 50 μg/ml of propidium iodide (PI) containing 0.25 mg/ml of RNase A at room temperature for 30 min. In addition, AGS cells were washed twice with ice‐cold, phosphate‐buffered saline and incubated in 5 μl of annexin V fluorescein isothiocyanate (FITC) and 5 μl of PI at room temperature in the dark for 15 min. Analyses were performed using a FACSCaliburTM instrument equipped with CELLQuestTM software (Becton Dickinson). 1 × 10^4^ cells (events) analysed by flow cytometry in each condition. Each determination was repeated three times. Acetaldehyde dehydrogenase (ALDH) in both AGS cells and culture media after mAb 201E4 treatment were determined by ALDH activity detection kit (boxbio) and calculated according to the instructions. The productions of 1 nmol NADH/min in 10^4^ cells and 1 ml culture media is defined as 1 enzyme activity unit, respectively (U/10^4^ cells and U/ml, respectively).

### 2.5 Western‐Blotting (WB)

1 × 10^6^ cells were treated with or without 20 μg/ml of mAb 201E4, harvested, and resuspended in PBS. After centrifugation at 200 *g* for 5 min, the pellet was lysed in ice cold P‐MER cell lyse buffer containing 1% Halt Protease and Phosphatase Inhibitor Cocktail (Thermo Scientific) for 30 min. The supernatant was collected after 10 min of centrifugation and stored at 4°C. Western blot analyses were carried out according to standard protocols. Immunoblots were developed using ECL chemiluminescence reagent (Thermo Scientific).

### 2.6 Apoptosis pathway activities

1 × 10^6^ AGS cells were treated with the 201E4 mAb (20 μg/ml) and collected at 0, 12, 24, and 36 h after mAb 201E4 incubation. Cell lysate proteins (50 μg per lane) were loaded on SDS‐PAGE, transferred to PVDF, and probed with specific Abs, such as anti‐caspase‐8, anti‐cleaved‐caspase‐8/9 and anti‐Bak (Immunoway); anti‐caspase‐3, anti‐cleaved‐caspase‐3, anti‐PARP, anti‐AKT, anti‐pAKT, anti‐p44/42, anti‐p‐p44/42, anti‐p70 S6, anti‐PI3K p85, anti‐p53, anti‐Bcl‐XL and anti‐GAPDH (Cell Signalling Technology, USA), anti‐β‐action (ABclonal). All antibodies were diluted in 1% BSA‐TBST.

### 2.7 Enzyme‐linked immune sorbent assay (ELISA)

The experiment was based on the double antibody sandwich‐ELISA. After tumour cell lysis, the polyclone antibody (Santa Cruz) against DR3, DR4, DR5, FAS‐R, TNFR1, TNFR2, transferrin‐receptor, CD147, ATP1A2, ICAM‐1, integrin beta 5, SLC3A2, etc., were coated for 2 h at room temperature. Following three times washing, the 96‐well plates were blocked with 1% BSA in PBS at room temperature for 2 h. The cells lysates were added and incubated for 2 h at room temperature. After washing, mAb 201E4 was added at a concentration of 1 μg/ml and incubated for 1.5 h at room temperature. Following washing, the wells were incubated with horseradish peroxidase‐labelled anti‐mouse IgG (Santa Cruz) for 1.5 h at room temperature. Finally, unbound proteins were removed by washing, substrate 3,3′‐5,5’‐Tetramethylbenzidine (TMB) (IDEXX Laboratories, Inc) was used for colour development at 37°C for 10 min, and the end point was read at 450 nm.

### 2.8 PNGase F and chemical reagents deglycosylation

Swainsonine (Sw) (a potent inhibitor of various α‐mannosidases) and Tunicamycin (Tm) (a N‐acetylglucosamine transferases inhibitor) (Cayman Chemical Company) were used to treat AGS cells in different concentrations. Sw was diluted 100 times (final concentration: 10 μg/ml), and Tm was diluted 400 times (final concentration: 2.5 μg/ml) as stock solution. The stock solution was added into the culture media according to the ratio of 1:9. After 5 × 10^3^ AGS cells were treated with Sw or Tm for 24 h, the Cy3 labelled mAb 201E4 was added to Sw‐ or Tm‐ treated cells for 6 h and the results were observed under the fluorescence microscope. 1 × 10^6^ AGS cells treated with Sw and Tm were collected and proteins were extracted for WB analysis. Cell lysates were deglycosylated using PNGase F (New England Biolabs) based on the manufacturer's instructions. Simply, 2 μg of cell lysates and 1 μl 10× Glycoprotein Denaturing Buffer and H_2_O (if necessary) were combined to make a 10 μl total reaction volume. Denatured glycoprotein was created by heating reaction at 100°C for 10 min. This was transformed into a total reaction volume of 20 μl by adding 2 μl 10× G7 reaction buffer, 2 μl 10% NP40, H_2_O and 1‐2 μl PNGaseF. The reaction was then incubated at 37°C for 1 h. The deglycosylated samples were analysed by WB.The vitality of Tm treated AGS cells were determined after mAb 201E4 treatment by CCK‐8 assay. Briefly, 5 × 10^3^ AGS cells were seeded into each well of 96 well plate and treated with 0.1 μg/ml Tm for 24 h (treatment group), while AGS cells without any treatments were as control (control group). Then, these cells in treatment group and control group were treated with mAb 201E4 (50 μg/ml) and with non‐specific mouse IgG, respectively. The absorbance was measured at 0, 24, 30, 54, and 78 h.

### 2.9 Statistical analysis

All data were analysed by anova (Version 19, IBM SPSS Statistics). *p*‐values < 0.05 were considered statistically significant. Data from the all assays are presented as mean ± standard deviation. Differences between two groups were examined using an unpaired *t*‐test or Mann–Whitney test. Comparisons among multiple groups were performed using one‐way anova and a post hoc test. Gene Ontology term analysis of the main gene expression was dot‐plotted through clusterProfiler packages in R language.

## 3 RESULTS

### 3.1 Reactivity characteristics of mAb 201E4 to cancers

**FIGURE 1:**
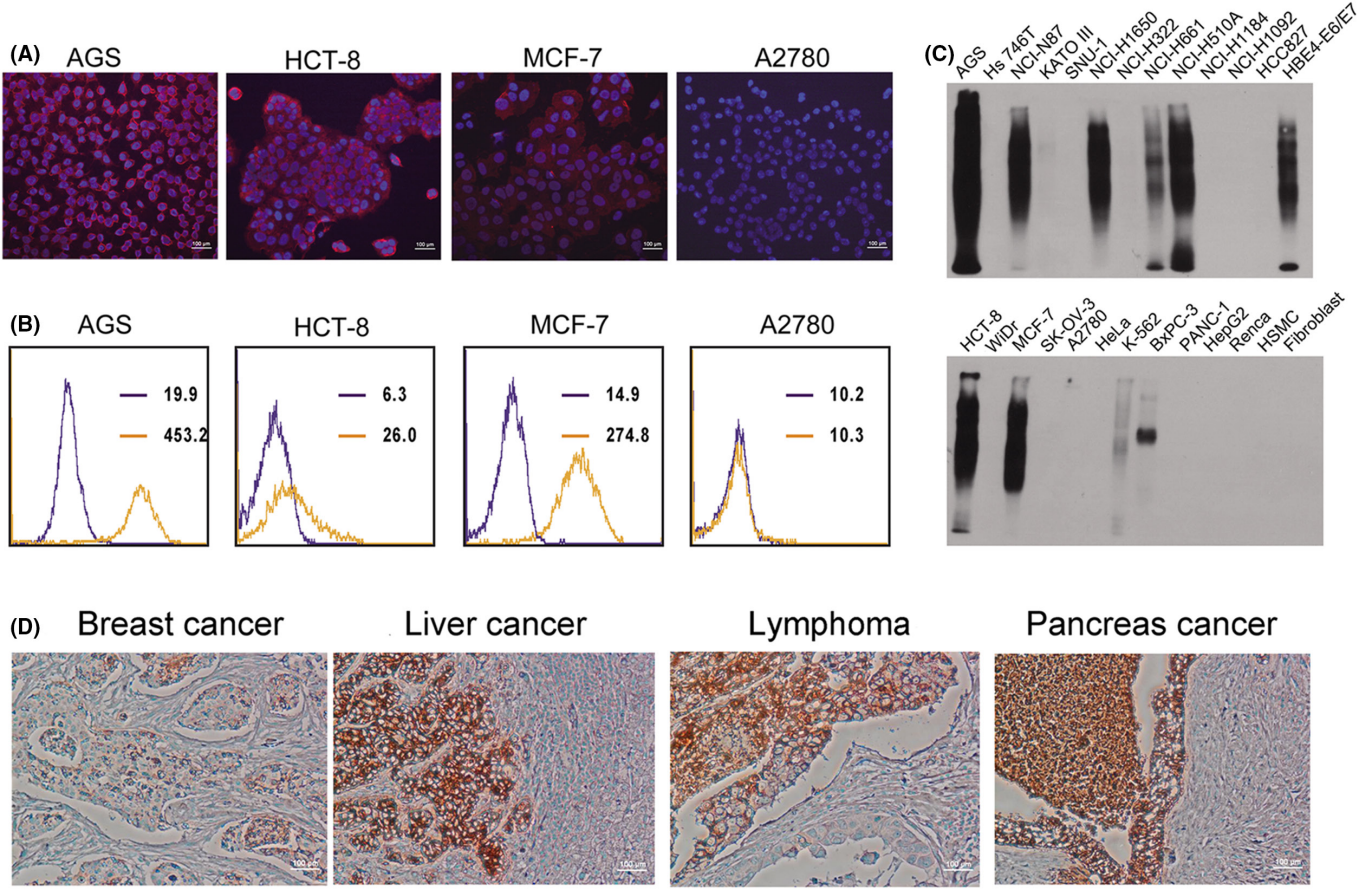
Reactivity characteristics of mAb 201E4 to cancers. (A) Cancer cell were screened by IFA using mAb 201E4 (100×). Red: goat anti‐mouse CY3‐labelled secondary antibody; Blue: Hoechst. (B) Cancer cells were analysed by FACs using mAb 201E4. 10^4^ cells (events) were acquired per sample with fluorescence measured on logarithmic scales in FACs. Cells were incubated with 2 μg/ml of mAb 201E4. (C) Reactivity of cell lysates with mAb 201E4 in WB. PVDF membranes were incubated with 1 μg/ml of mAb 201E4. (D) Reactivity of cancer tissues with mAb 201E4 in IHC (100× magnification). Tissues were incubated with 1 μg/ml of mAb 201E4.

The reactivity of mAb 201E4 to various cancer cells and normal cell lines were screened using immunofluorescence assay (IFA), fluorescence‐activated cell sorting (FACs), and western blot (WB) (Figure 1A–C). AGS, HCT‐8, and MCF‐7 strongly reacted with mAb 201E4, while A2780 did not react in IFA and FACs assays (Figure 1A,B). Other cancer cell lines which strongly reacted with 201E4 mAb included NCI‐N87, NCI‐H1650, and NCI‐H510A. Cancer cells lines NCI‐H661, K562, and BxPC‐3 were weakly identified by mAb 201E4. Cancer cell lines with no reactivity to mAb 201E4 included KATO III, SNU‐1, and HeLa (Figure 1C). It is interesting that unlike other mAbs, the band of protein for mAb 201E4 was represented by proteins of numerous sizes rather than a single or few bands that would be expected for a traditional protein bound mAb. This suggests mAb 201E4 recognized a specific structural molecule on many proteins rather than an isolated protein. A possible molecular weight range for a lot of proteins recognized by mAb 201E4 was from 55 KDa to 220 KDa according to main lanes in WB analysis, including <5 KDa few proteins. Finally, on IHC analysis of tumour tissue, mAb 201E4 was able to specifically identify cancer cells without binding to adjacent normal cells (Figure 1D).

### 3.2 The mAb 201E4 was directly cytotoxic to tumour cells in vitro and evoked tumour cell death

**FIGURE 2:**
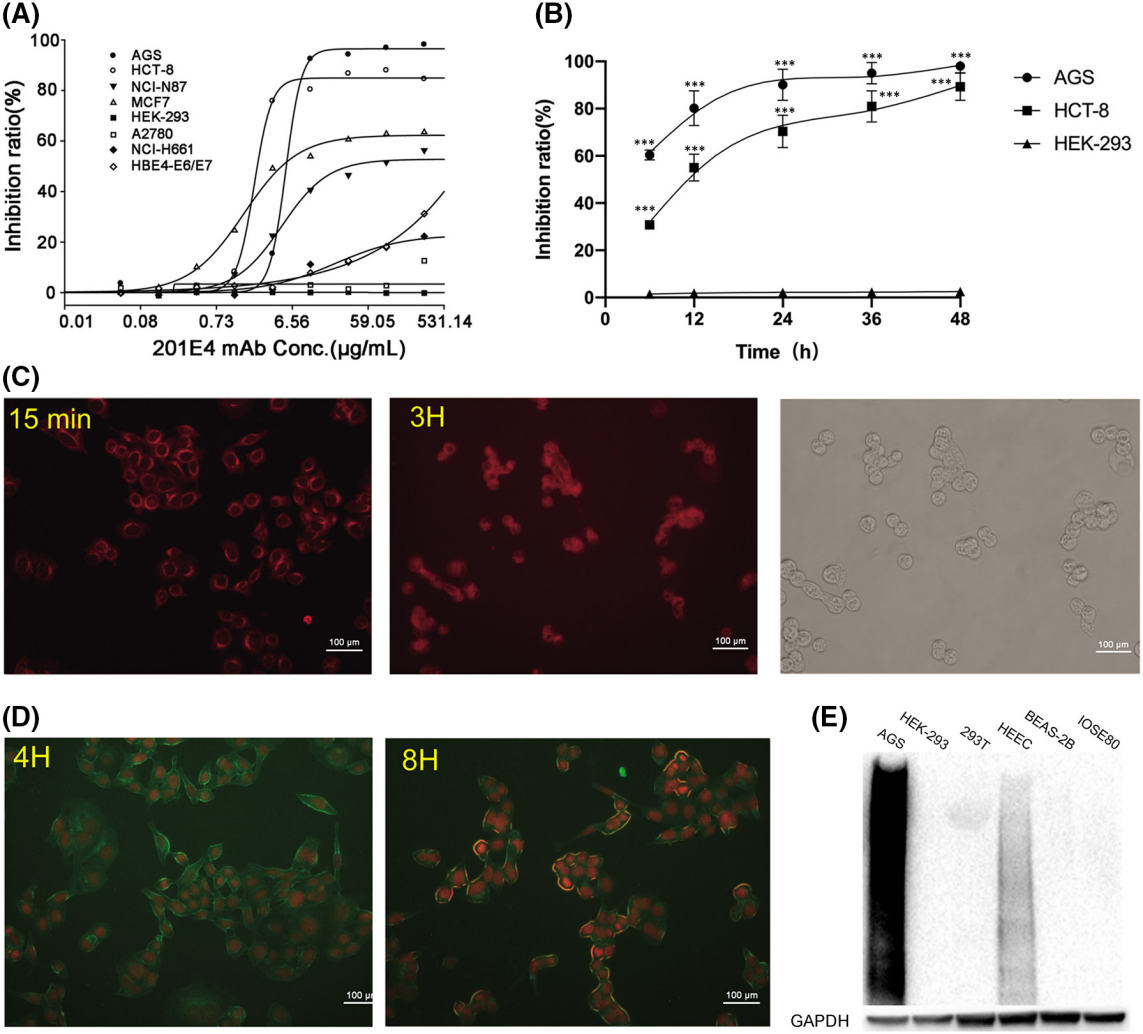
The mAb 201E4 inhibits cancer cells growth. (A) Cell cytotoxicity was measured using a CCK‐8 based assay. (B) Inhibition ratios of cells increased in a time‐dependent manner after 20 μg/ml of mAb 201E4 treatment. HEK‐293 is a non‐cancerous cell line and as the control. ***p < 0.001 versus the control. (C) Cy3‐labelled mAb 201E4 was able to bind to AGS cells in 15 min resulting in membrane destabilization. By 3 h, the majority of cells were dead (100×). Red: Cy3‐labelled mAb 201E4. (D) 2 μg/ml of FITC‐labelled mAb 201E4 was able effectively break the membrane of AGS celss within 8 h (100×). Green: FITC‐labelled mAb 201E4, Red: API staining. (E) Reactivity of common normal cell lysates with mAb 201E4 in WB. PVDF membranes were incubated with 1 μg/ml of mAb 201E4.

Anti‐tumour activity of mAb 201E4 against cancer cells was evaluated in vitro using the CCK‐8 assay. AGS and HCT‐8 cells appeared to be the most sensitive to mAb 201E4 with IC_50s_ of 2.77 and 1.49 μg/ml, respectively. MCF‐7 and NCI‐N87 cells were less sensitive with IC_50s_ of 5.10 and 41.22 μg/ml, respectively. NCI‐H661 was slightly inhibited by mAb 201E4, while A2780 was not sensitive to mAb 201E4 (Figure 2A). There appeared to be a correlation between maximum inhibition ratio and the associated reactivity of a given cell line in WB. AGS had a close prefect maximum inhibition ratio at almost 100%, while HCT‐8, MCF‐7, NCI‐N87 were 88%, 64%, and 56%, respectively. Time correlation of the inhibition ratio showed mAb 201E4 have rapid inhibition effect beginning from 12 h of mAb 201E4 treatment, with the inhibition ratio of about 80% (AGS) and 55% (HCT‐8), respectively (Figure 2B).

The antibody was highly effective at eliminating AGS cells in culture assay (Figure 2C,D). Surprisingly, Cy3‐labelled mAb 201E4 was able to bind to AGS cell membranes at 15 min and break or cause cell destruction within 3 h of mAb 201E4 treatment (Figure 2C). Even using 2 μg/ml of mAb 201E4 was able to effectively break the membrane of AGS cells at 8 h of 201E4 treatment, but the nucleus had little change over the same time frame (Figure 2D).

The effects of mAb 201E4 on normal cells were investigated by the immunoblot and cytotoxicity assay, such as HBE4‐E6/E7, HEK‐293, HSMC, Fibroblast, 293T, BEAS‐2B, HEEC and IOSE80. Most normal cell lines had either weak or no reaction to mAb 201E4, except HBE4‐E6/E7 in immunoblot analysis (Figures 1C and 2E). Meanwhile, HBE4‐E6/E7 was slightly sensitive to mAb 201E4 with the increase of concentrations (Figure 2A), while HEK‐293 was not sensitive (Figure 2A,B). These results suggested that the growth of cells with antigenic determinants recognized by 201E4, including cancer cells and normal cells, may be inhibited by mAb 201E4. However, only few normal cell lines are reactive with mAb 201E4. After all, most cancer cell lines are more sensitive to mAb 201E4, while HBE4‐E6/E7 is only sensitive at high concentration of mAb 201E4.

### 3.3 The mAb 201E4 inhibited tumour growth in xenografts model

**FIGURE 3:**
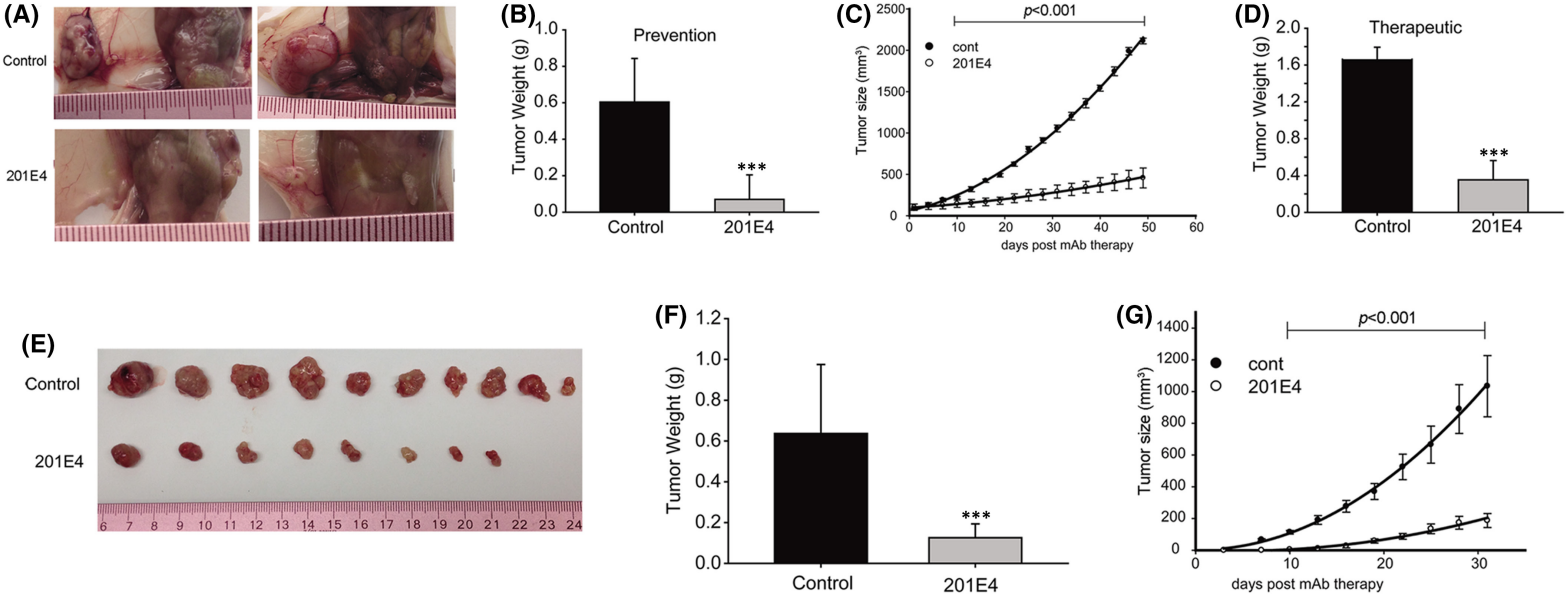
Animal models of mAb 201E4 to cancer cells. (A–D) AGS cells xenograft model (*n* = 6). (E–G) HCT‐8 cells xenograft model (*n* = 10). (A) Photographs of tumours from the prevention group and control group in AGS xenograft. (B) Tumour weight recorded after the mice were sacrificed at 30 days in prevention group. ***p < 0.001 versus the control. The prevention group was treated with mAb 201E4 (25 mg/kg/p3d) immediately after AGS cells inoculation. The same concentrations of non‐specific mouse IgG were used in control mice. (C) Effect of mAb 201E4 (25 mg/kg/p3d) on growth of tumour xenograft in therapeutic group. Tumour growth was tracked by the mean tumour volume (mm^3^) ± SD. (D) Tumour weight recorded after the mice were sacrificed at 50 days in therapeutic group. The therapeutic group consisted of treatment with mAb 201E4 (25 mg/kg/p3d) at day 14 post inoculation of AGS cells when the implanted tumour had grown to 50–100 mm^3^. ***p < 0.001 versus the control. (E) Photographs of tumours from the prevention group and the control in HCT‐8 xenograft. (F) Tumour weight recorded after the mice were sacrificed at 33 days in prevention group. ***p < 0.001 versus the control. (G) Effect of mAb 201E4 (25 mg/kg/p3d) on growth of tumour xenograft in prevention group. Tumour growth was tracked by the mean tumour volume (mm^3^) ± SD. The prevention group was treated with mAb 201E4 (25 mg/kg/p3d) immediately after HCT‐8 cells inoculation. The same concentrations of non‐specific mouse IgG were used in control mice.

To evaluate the anti‐tumour activity of mAb 201E4 in vivo, the antibody was tested in two xenograft models. One using gastric cancer cells, AGS, and the other using colon cancer cells, HCT‐8. This was tested in male BALB/c nude mice. The mAb 201E4 significantly inhibited AGS tumour growth in both the prevention and therapeutic dosing arms from the first week up until sacrifice at 50 days (*p* < 0.001) (Figure 3A–D). No tumours were generated with 50% (3/6) of mice after treatment with mAb 201E4 in the prevention group within 30 days of treatment (Figure 3A). There was significant difference in tumour weight at 30 days between the prevention group and the control (*p* < 0.001, Figure 3B). The difference was also demonstrated visually in the therapeutic group (Figure 3C,D). The growth of AGS xenograft was significantly inhibited by mAb 201E4 in therapeutic group during 50 days of mAb 201E4 treatment (*p* < 0.001, Figure 3C). Tumour weight in therapeutic group with mAb 201E4 treatment was significantly lower than that of the control (*p* < 0.001, Figure 3D). Furthermore, in the xenograft model using HCT‐8 cells, treatment with mAb 201E4 also resulted in a significant reduction in tumour weight and tumour growth (*p* < 0.001) and total elimination of the tumour in 20% of mice (2 of 10) (Figure 3E–G).

### 3.4 Mechanism of mAb 201E4 induced apoptosis in AGS

**FIGURE 4:**
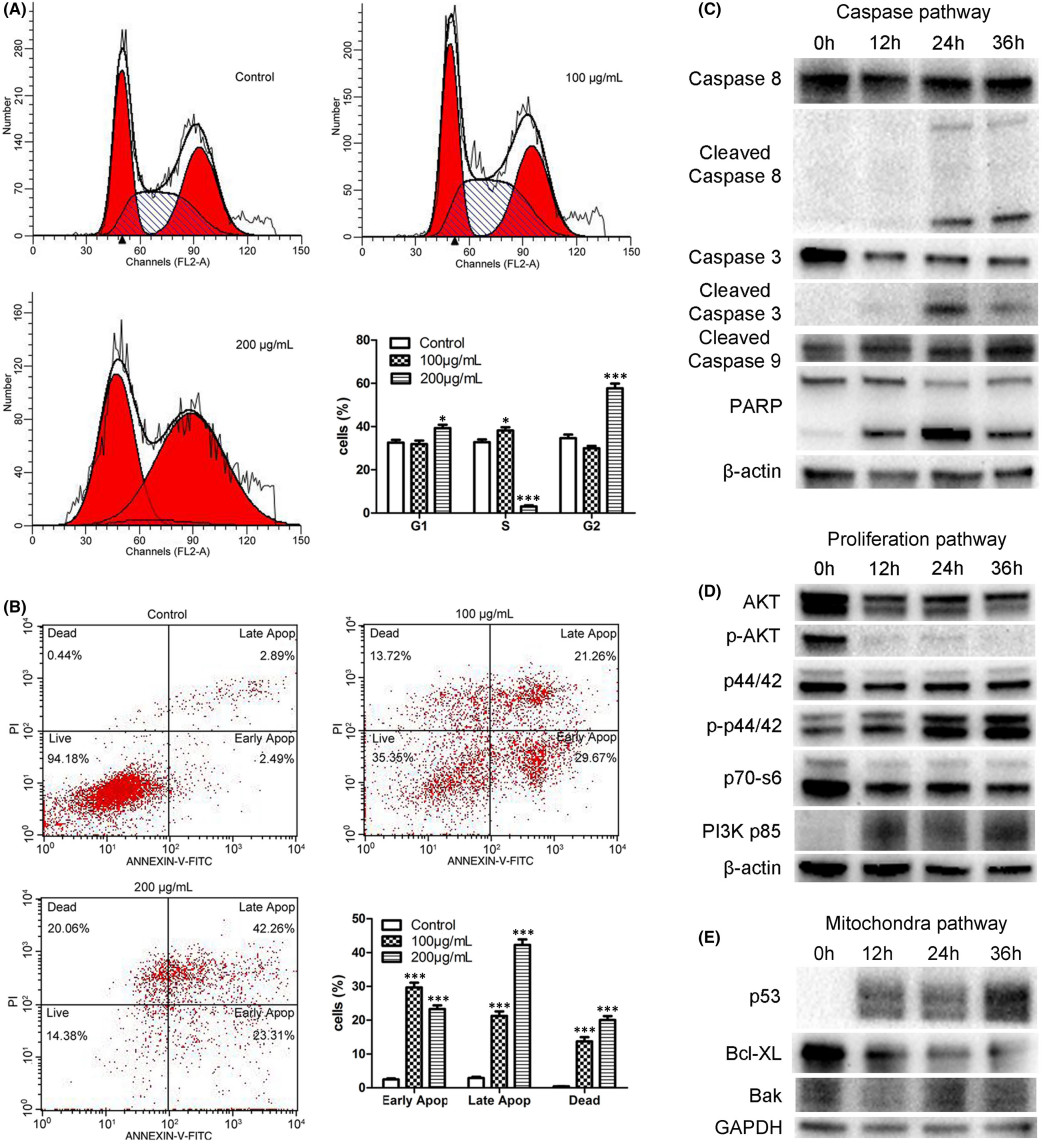
The mechanism of mAb 201E4 induced apoptosis in cancer cells. (A) Flow cytometry of cell cycle phase distribution and the percentages of cell cycle population were shown after 24 h of mAb 201E4 treatment (100 and 200 μg/ml) in AGS cells. Non‐specific mouse IgG was as a control. Data shown are the mean values of three independent experiments ±SD. *p < 0.05 versus the control. ***p < 0.001 versus the control. (B) mAb 201E4 induces apoptosis in AGS cells by flow cytometry. AGS cells were treated with mAb 201E4 (100 and 200 μg/ml) for 24 h. Apoptosis was monitored by Annexin V/PI double staining. Percentages of early apoptotic cells, late apoptotic cells, and dead cells in AGS treated with mAb 201E4 (100 and 200 μg/ml) were shown. Non‐specific mouse IgG was as a control. The values are means ± SD of three independent experiments. ***p < 0.001 versus the control. (C–E) Time‐dependent effects of mAb 201E4 on the expression of proteins in the caspase apoptotic pathway, the proliferation pathway, the mitochondral apoptotic pathway in immunoblot analysis, respectively. AGS cells were treated with mAb 201E4 (20 μg/ml) at 0, 2, 6, 12, 24, and 36 h.

The cell cycle distribution of AGS cells was analysed after treatment with mAb 201E4 for 24 h. Indeed, marked G2 phase arrest was observed in AGS cells treated with the mAb (Figure 4A). The percentage of the cell population in the G1 phase was increased compared to that of the control in a dose‐dependent manner (*p* < 0.05). At a treatment with 200 μg/ml of mAb 201E4, the S phase population were dramatically decreased (*p* < 0.001). However, at the 100 μg/ml concentration, there was a slight increase in S phase (*p* < 0.05).

Because cell cycle arrest plays an important role in apoptosis of tumour cells, the abilities of mAb 201E4 to induce cell apoptosis or death in AGS cells was further investigated by FACs. The effects of different concentrations of mAb 201E4 on AGS cells were compared by Annexin V/PI staining. The Annexin V positive cells were regarded as early apoptotic cells (AV+/PI−) or late apoptotic cells (AV+/PI+). In comparison to the isotype control, the late apoptotic ratios of AGS cells treated with 100 and 200 μg/ml of mAb 201E4 were increased in a dose‐dependent manner, to 21.26% and 42.46%, respectively (*p* < 0.001, Figure 4B). Moreover, the necrotic ratios of AGS cells also increased in a dose‐dependent manner after treatment (*p* < 0.001). Compared to the control, the early and late apoptotic ratios of AGS cells were also increased (*p* < 0.001). The increased dead and apoptotic AGS cells resulted in reduced numbers of living AGS cells. In addition, ALDH in both AGS cells and culture media after mAb 201E4 treatment significantly decreased in comparison to that of the control, in a dose‐dependent manner, respectively (*p* < 0.001, Figure S1A,B). Especially, ALDH trended to 0 U/ml in culture media of AGS cells treated with 100 and 200 μg/ml of mAb 201E4, suggesting the productions of NADH had been virtually blocked (Figure S1B). These support that mAb 201E4 induces AGS cell apoptosis and necrosis effectively.

In order to further characterize the apoptotic mechanism of mAb 201E4, caspase, proliferation, and mitochondrial apoptosis‐associated pathway proteins were detected by WB (Figure 4C–E). Cleaved caspase‐3, cleaved caspase‐8 and cleaved caspase‐9 were highly activated in a time‐dependent manner, suggesting that the caspase pathway was activated by mAb 201E4 treatment (*p* < 0.01, Figure 4C). Moreover, caspase‐8 was highly activated in AGS cells treated with mAb 201E4, suggesting that the Fas‐mediated apoptosis pathway was also activated by treatment. The results of the caspase pathway activation were consistent with the increase in the necrotic and apoptotic ratios of AGS cells treated with mAb 201E4 noted in the PI staining (Figure 4B). This indicates that the activation of caspases is a crucial pathway of apoptosis in AGS cells treated with mAb 201E4.

The expressions of PI3K p85 and p–P 44/42 proteins was also increased in AGS cells treated with mAb 201E4. Although the expression of AKT protein was relatively stable, the expression of p‐AKT protein decreased, suggesting the phosphorylation level was decreasing after AGS cells were treated with mAb 201E4 (Figure 4D).

Furthermore, the expression of Bak protein, a necessary signal to initiate apoptosis, began to increase in AGS cells from 24 h of mAb 201E4 treatment. Bcl‐XL, one of the antiapoptotic members of the Bcl‐2 family, maintained high levels at 12 h of mAb 201E4 treatment and decreased from 12 to 36 h of mAb 201E4 treatment. As an important tumour suppressor, p53 plays a protective role in many cellular processes that are capable of activating multiple target genes, leading to cell cycle arrest. The expression levels of p53 in AGS cells treated with mAb 201E4 were significantly increased from 12 to 36 h of mAb 201E4 treatment (Figure 4E).

### 3.5 Anti‐tumour activity of mAb 201E4 is associated with glycosylation in cancer cells

**FIGURE 5:**
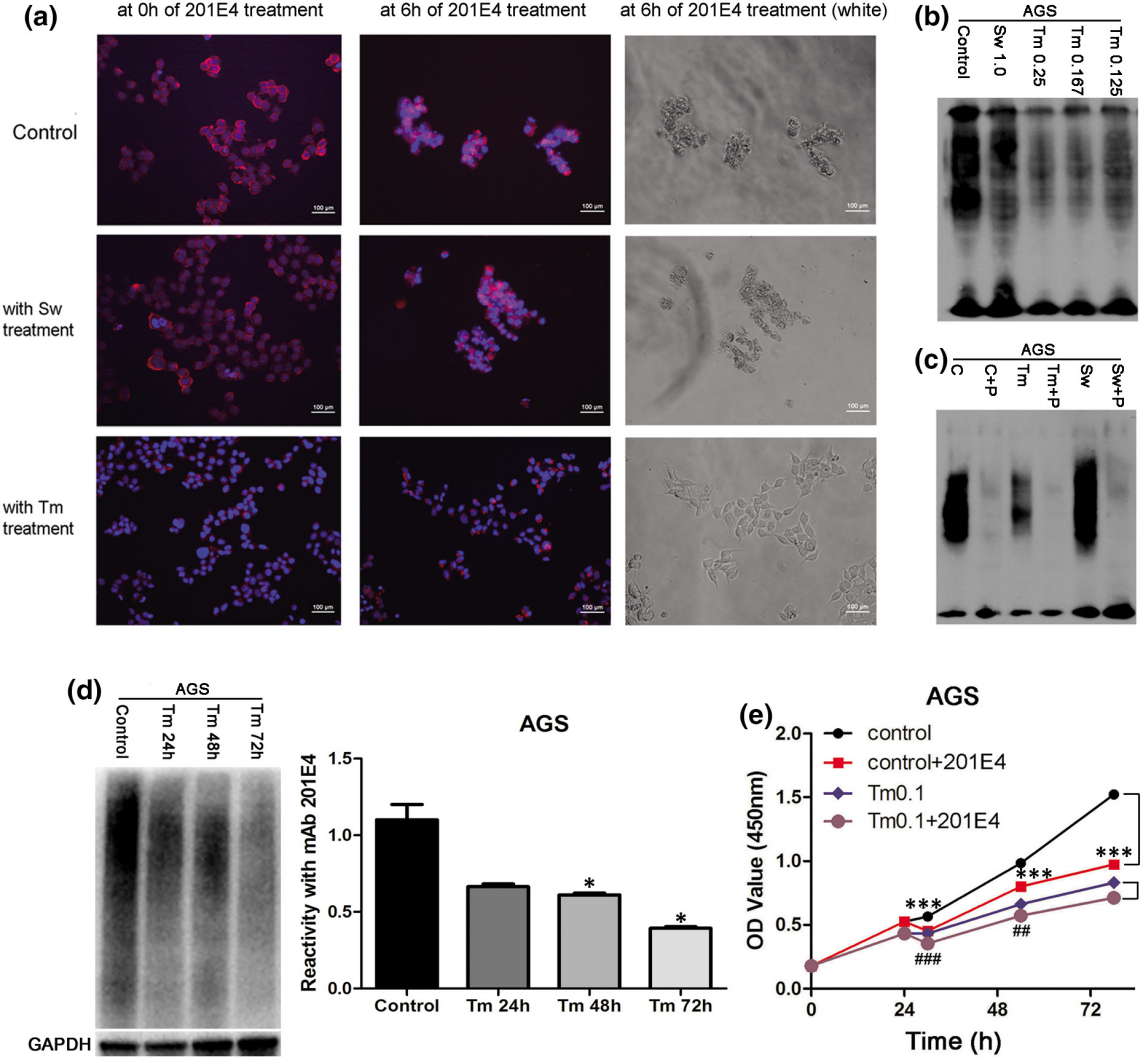
The deglycosylation analysis of mAb 201E4. (A) AGS cells were treated with Sw (1.0 μg/ml) and Tm (0.25 μg/ml) for 24 h. Then, Sw‐ or Tm‐treated AGS cells were treated with 20 μg/ml of mAb 201E4 for 6 h. The control AGS cells did not receive any treatments. The Tm‐treated AGS cells were weakly recognized by mAb 201E4 at 24 h of Tm‐treatment and survived after 6 h of mAb 201E4 treatment (100×). Red: Cy3‐labelled mAb 201E4; Blue: Hoechst. (B) Reactivity of 201E4 with AGS cells inhibited by glycosylation (Sw and Tm). AGS cells were treated with 1 μg/ml of Sw and 0.25, 0.167, 0.125 μg/ml of Tm. The lysate of AGS cells without any treatments was as a control in WB. (C) Reactivity of 201E4 with AGS cells lysates deglycosylated by PNGase F. Deglycosylation of cell lysates were performed by PNGase F (2 μl). (C) The lysate of AGS cells without any treatments was as a control; C+P: The lysate of AGS cells with PNGase F treatment; Tm: The lysate of Tm‐treated AGS cells; Tm+P: The lysate of Tm‐treated AGS cells with PNGase F treatment; Sw: The lysate of Sw‐treated AGS cells; Sw+P: The lysate of Sw‐treated AGS cells with PNGase F treatment. AGS cells were treated with 1 μg/ml of Sw and 0.1 μg/ml of Tm. (D) Reactivity of 201E4 with AGS cells decreased in a time‐dependent manner after 0.1 μg/ml of Tm treatment. The lysate of AGS cells without any treatments was as a control. *p < 0.05 versus the control. (E) Cell proliferation was measured using a CCK‐8 based assay. Control: AGS cells without any treatments; Control+201E4: 50 μg/ml of mAb 201E4 was added when AGS cells grew for 24 h; Tm0.1: AGS cells treated with 0.1 μg/ml of Tm; Tm0.1+201E4: 50 μg/ml of mAb 201E4 was added when AGS cells treated with 0.1 μg/ml of Tm for 24 h. ***p < 0.001 versus the control. ##*p* < 0.01 versus Tm0.1. ###*p* < 0.001 versus Tm0.1. Sw, Swainsonine; Tm, Tunicamycin.

The α‐mannosidase inhibitor (Sw) and N‐acetylglucosamine transferase inhibitor (Tm) represent two kinds of glycosylation inhibitors. These were used to deglycosylate AGS cells. After AGS cells were treated with Tm, mAb 201E4 was had minimal binding to the cells and was unable to cause cell death after 6 h (Figure 5A). However, Sw treatment did not stop mAb 201E4 from binding or inducing cell death after 6 h (Figure 5A). These results were confirmed visually in Figure S2, which shows again that Tm treated AGS cells were unable to be killed by mAb 201E4, while controls and Sw treated cells were still susceptible.

The lysates of AGS cells treated with Sw (1.0 μg/ml) and Tm (0.25, 0.167, and 0.125 μg/ml) were detected by WB using mAb 201E4 (Figure 5B). Cell lysates of the control and Sw‐treated AGS cells were able to be recognized by mAb 201 E4. However, there was minimal recognition of proteins in the Tm (0.25 μg/ml) treated lysate. The possible reason for the the weak fluorescence of the Tm‐treated lysate was likely related to there being some residual activity of the N‐acetylglucosamine transferase enzyme.

The lysates of AGS cells treated with Sw (1.0 μg/ml) and Tm (0.25 μg/ml) were further deglycosylated by PNGase F. After deglycosylation by PNGase F, mAb 201E4 was unable to recognize any proteins within the lysate of the control, Sw‐treated or Tm‐treated AGS cells (Figure 5C). This further supported that the antigen recognized by 201E4 mAb was associated with glycosylation rather than a specific protein.

To further verify that mAb 2014 E4 targeting a product of glycosylation, Tm‐treated AGS cells were continued to treat with mAb 201E4. The reactivities of lysate from Tm‐treated AGS cells (0.1 μg/ml) with mAb 201E4 significantly decreased at 48 and 72 h after Tm treatments compared with the control (*p* < 0.05, Figure 5D). These results indicated low degrees of glycosylation associated with N‐acetylglucosamine transferase was modified in Tm‐treated AGS cells. Similarly, the vitality of Tm‐treated AGS cells treated with mAb 201E4 was no lower than that in Tm‐treated AGS cells treated without mAb 201E4 at 78 h after Tm treatments (*p* > 0.05, Figure 5E). On the contrary, untreated AGS cells were extremely inhibited by mAb 201E4 at 78 h of growth (or at 54 h of mAb 201E4 treatment) compared with AGS cells without any treatments (*p* < 0.001, Figure 5E). These results clarified mAb 201E4 kills AGS cells through target of glycosylation associated with N‐acetylglucosamine transferase.

### 3.6 Targets and effects of mAb 201E4 on cancer cell transcriptome

**FIGURE 6:**
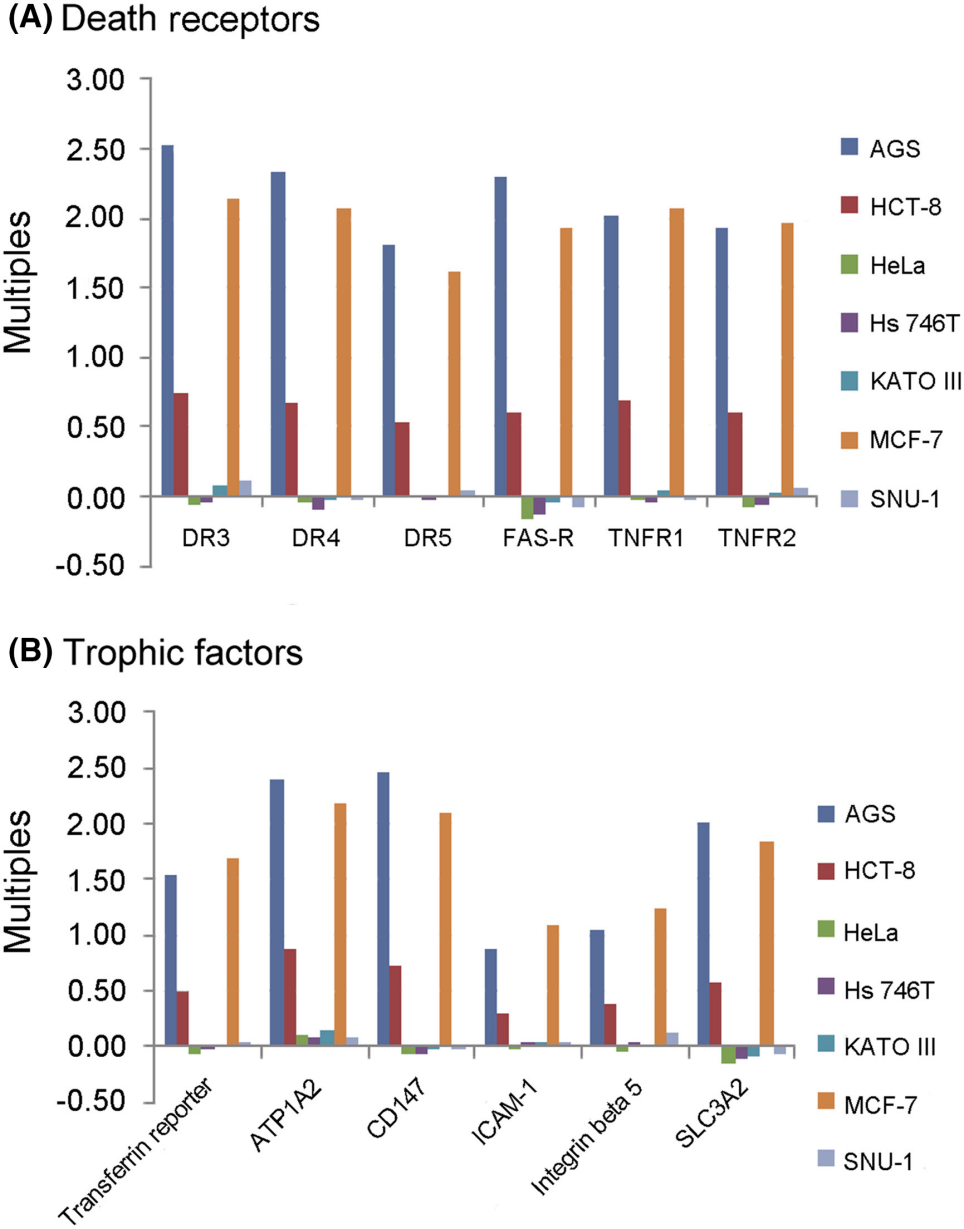
Parts of potential receptors and factors were identified by ELISA. According to the results of mass spectra analysis, parts of death receptors and trophic factors were chosen and detected by ELISA. The levels of death receptors and trophic factors which be identified by mAb 201E4 were high in some cancer cell lysate by sandwich‐ELISA, especially AGS, MCF‐7 and HCT‐8 cells. Cancer cells were treated with 20 μg/ml of mAb 201E4 for 72 h (treatment group). The lysate of cancer cells without any treatments was as a control (control). Multiples were OD_450_ of treatment group/OD_450_ of control.

To assess what receptors mAb 201E4 maybe binding to induce cancer cell destruction, death receptors and trophic factors were analysed by ELISA (Figure 6). These receptors and factors were high in AGS, HCT‐8 and MCF‐7 cells (Figure 6) which were known to be susceptible to mAb 201E4 based on prior experiments (susceptible Figure 2A). Based on this, it was hypothesized that mAb 201E4 may work through binding glycans on the upregulated death receptors or trophic factors. To confirm these were potential antigens recognized by mAb 201E4, mass spectra was performed on immunoprecipitated AGS lysate. Many functional enzymes and proteins were noted by mass spectra, including ATPases, transferases, receptors, transporters, integrins, adhensions, and cadherins (*p* < 0.001) (Table S1). To analyse the effect of mAb 201E4 on destroyed AGS cells, transcriptome sequencing was performed using AGS cells after mAb 201E treatment using KEGG pathway classification and Gene Ontology enrichment analysis (Figures S3 and 4). These results indicated that multiple genes and pathways were involved in the process of AGS cell induced killing or apoptosis by mAb 201E4 associated with glucoside.

## 4 DISCUSSION

Monoclonal antibodies, such as pembroluzimab and transtuzmab, have drastically changed the face of cancer treatment. These mAbs offer the ability to target a specific protein and often have better side effect profiles compared to traditional chemotherapy. However, these antibodies do not directly cause cancer cell death; instead, they often rely on other effectors to terminate cancer cells.^17^, ^18^, ^19^, ^20^ The cancer can develop resistance to these effectors through modifying its environment or acquiring mutations.^6^ Furthermore, these anticancer mAbs only recognize extracellular or cell surface proteins, which constitute a small fraction of the cellular proteins and may not be tumour‐specific.^21^, ^22^, ^23^, ^24^, ^25^, ^26^ Therefore, antibodies which are able to cause cell death on binding and target both intracellular and extracellular proteins may offer improved efficacy.

Based on this, glycans are an ideal target as these modifications are present on numerous proteins.^27^, ^28^ In fact, few glycan mAb therapy is currently being investigated in clinical trials. In this study, the mAb 201E4, which targeted specific glycans, possessed numerous characteristics, which make it a promising candidate to potentially advance to use in a clinical setting.

First, mAb 201E4 targets a specific glycosylation product. This was demonstrated by the antibody not binding any cell lysate after treatment with PNGase F, an agent which causes deglycosylation indiscriminately. The antibody showed different anti‐tumour activity when AGS cells were treated with a α‐mannosidase inhibitor or a N‐acetylglucosamine inhibitor, which indicates its specificity for particular glycans. These particular glycans which can be recognized by mAb 201E4 play an important role in cell death induced by mAb 201E4. This suggests that mAb 201E4 maybe kills cancer cells through targeting certain glycosylation products on pathways.

While glycosylation is a common process in all cells, mAb 201E4 seemed to have preferential binding to cancer cells. Indeed, this was demonstrated by the lack of binding of the mAb to HSMCs or fibroblasts. Immunohistochemistry also showed that mAb 201E4 would bind to cancerous cells but not to adjacent normal cells. However, the mAb did bind to HBE4‐E6/E7, which is a lung cell line transformed with HPV. It is unclear whether this represents a reaction to normal tissue or changes caused by the HPV virus which promoted abnormal glycosylation facilitating antibody binding. Nonetheless, these findings further support that the tested mAb preferentially binds aberrant glycosylation modifications that are cancer specific, with minimal reaction to normal tissues.

Different from most currently available mAbs, mAb 201E4 was able to induce cell death without effector cells. The mechanism seemed to be related to both membrane destabilization and apoptosis pathways. This may be due to that the mAb was able to bind to numerous proteins, which is a very rare phenomenon. These findings are not unexpected given it is thought that more than 50% of eukaryotic proteins are modified by glycosylation. Aberrant display of the truncated core1 O‐glycan T‐antigen is a common feature of human cancer cells that correlates with metastasis.^29^

Finally, the antibody was tested in vivo where it continued to highly effective as demonstrated by the reduction in tumour weight and prolonged survival of treated mice in both the prevention and therapeutic dosing regimens. This was particularly exciting as prevention dosing completed inhibited tumour growth in 50% of mice. The findings of the prevention group as it indicates this antibody maybe a tremendously effective maintenance therapy to specifically target and destroy residual microscopic disease after primary treatment.

In the present study, mAb 201E4 was able to successfully target glycan modifications. It also demonstrated the hallmarks of a successful cancer treatment. These included having a specific target, inducing cell death, and having minimal off target effects. Furthermore, mAb 201E4 was successful in an in vivo model. In summary, because of these advantageous attributes, mAb 201E4 may provide a new strategy for antibody therapy in cancer.

## AUTHOR CONTRIBUTIONS

**Heng Xu:** Conceptualization (equal); data curation (equal); formal analysis (lead); writing – original draft (equal). **Boying Dun:** Methodology (lead); project administration (lead); validation (equal); writing – original draft (equal). **Beiyi Liu:** Methodology (supporting); resources (lead); validation (equal). **David Mysona:** Writing – review and editing (equal). **JIn‐Xiong She:** Conceptualization (equal); data curation (equal); writing – review and editing (equal). **Rong Ma:** Data curation (equal); formal analysis (equal); funding acquisition (equal).

## CONFLICT OF INTEREST

The authors declare that they have no conflict of interest.

## Supporting information


Figure S1

Figure S2

Figure S3

Figure S4

Table S1

## Data Availability

The data that support the findings of this study are available from the corresponding author upon request.
