# Supplementary figures and images for: A novel monoclonal antibody associated with glucoside kills gastric adenocarcinoma AGS cells based on glycosylation target
Source: J Cell Mol Med. 2022 Aug 9;26(18):4781–91. doi: 10.1111/jcmm.17504 (PMC9465190; doi:10.1111/jcmm.17504)

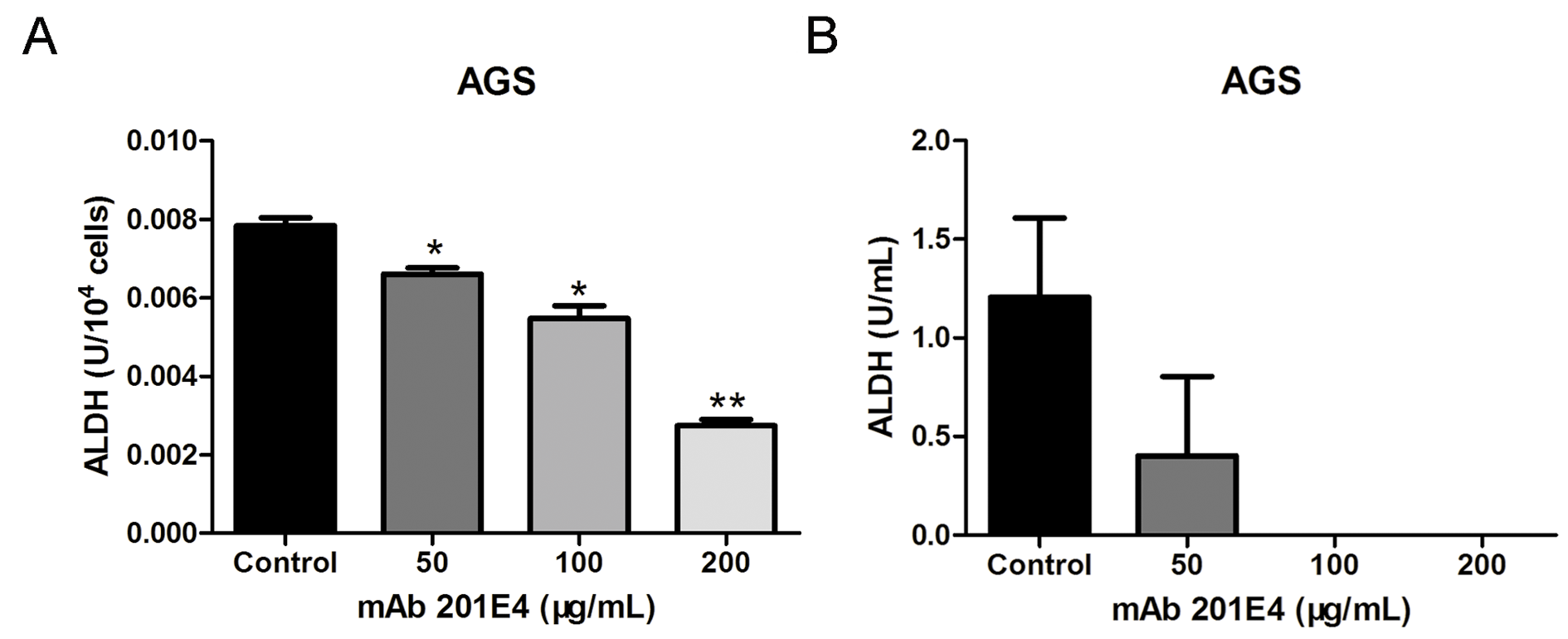

Supplement: Supplementary file 1 — Figure S1 [file JCMM-26-4781-s005.tif]

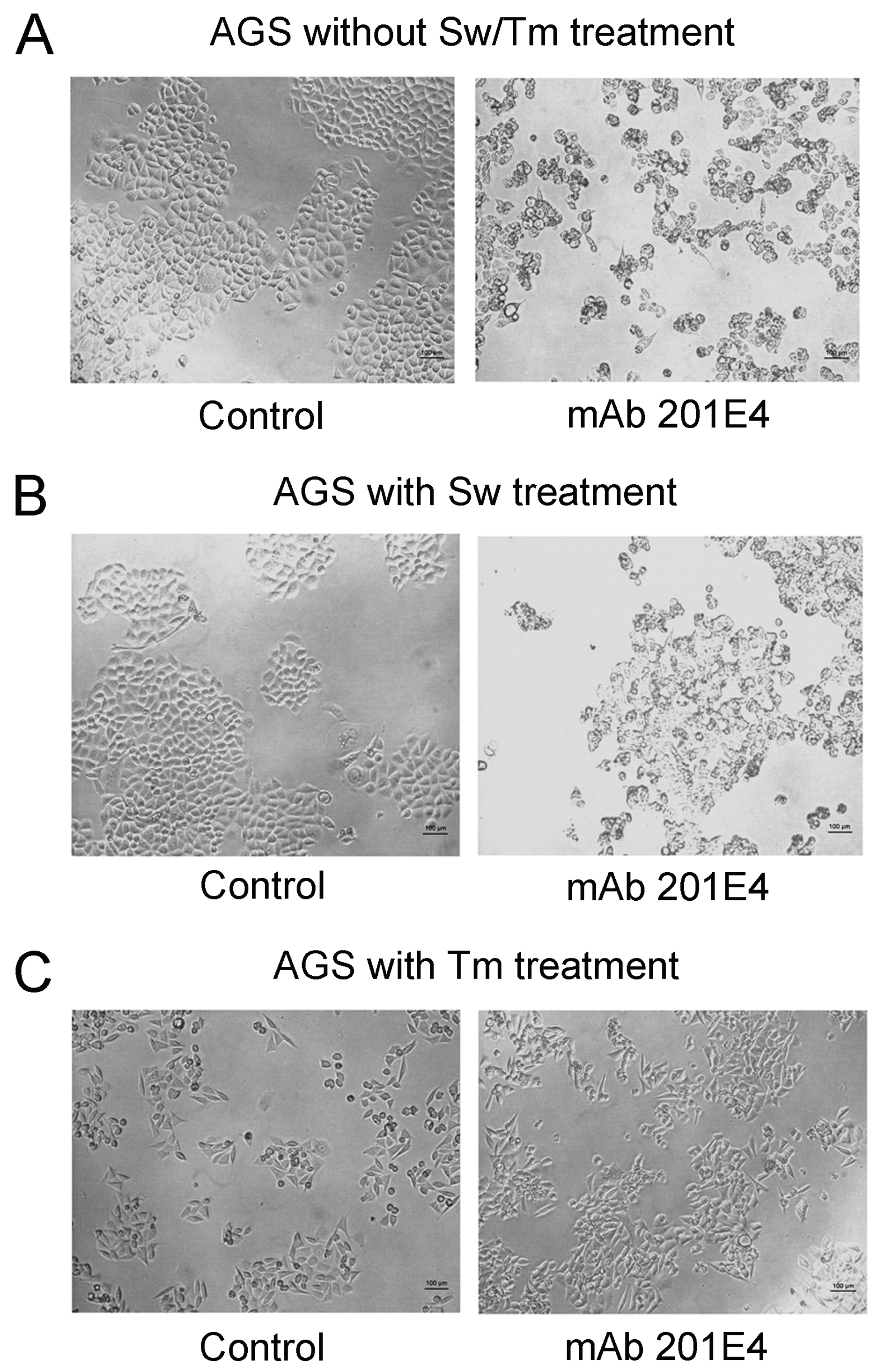

Supplement: Supplementary file 2 — Figure S2 [file JCMM-26-4781-s001.tif]

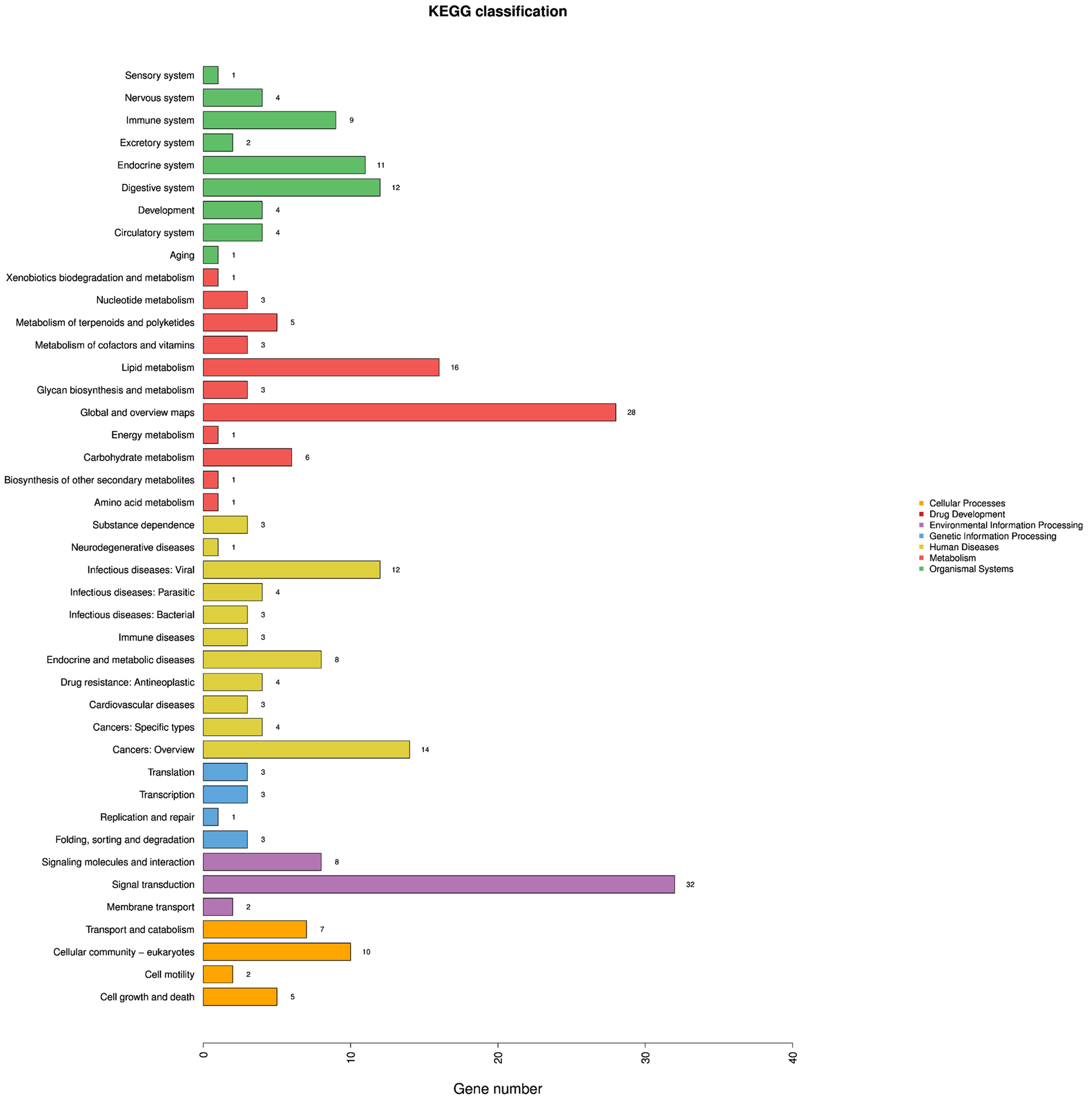

Supplement: Supplementary file 3 — Figure S3 [file JCMM-26-4781-s004.tif]

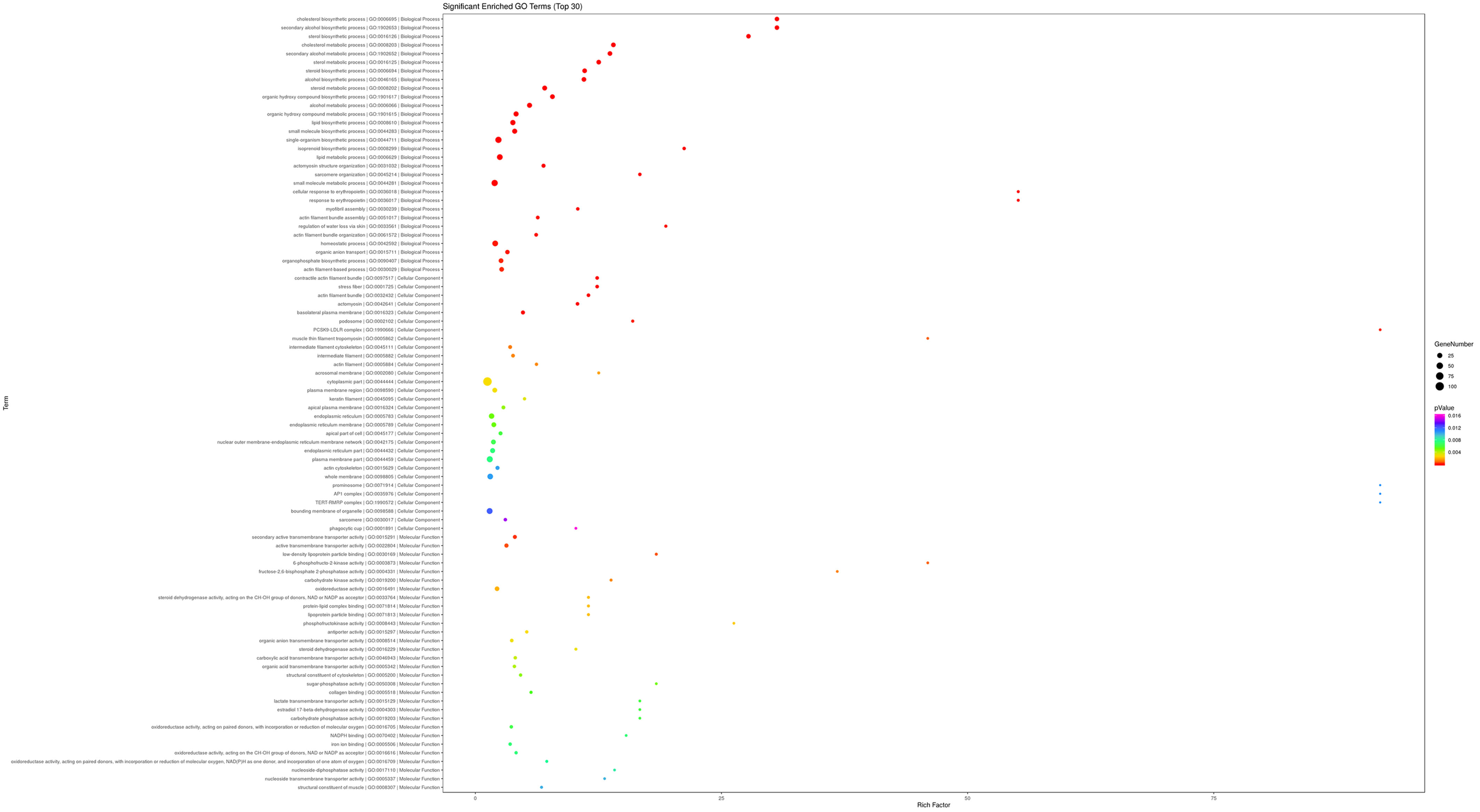

Supplement: Supplementary file 4 — Figure S4 [file JCMM-26-4781-s003.tif]
