# Supplementary material for: A novel monoclonal antibody associated with glucoside kills gastric adenocarcinoma AGS cells based on glycosylation target
Source: J Cell Mol Med. 2022 Aug 9;26(18):4781–91. doi: 10.1111/jcmm.17504 (PMC9465190; doi:10.1111/jcmm.17504)
Supplement: Supplementary file 5 — Table S1 [file JCMM-26-4781-s002.docx]

**Supplementary Table**

Table S1 Mass spectra results

| **Function** | **Peptide** | **P value** |
| --- | --- | --- |
| ATPase | Gene_Symbol=ATP1A1 Isoform Long of Sodium/potassium-transporting ATPase subunit alpha-1 | 3.46E-10 |
|  | Gene_Symbol=ATP1A2 Sodium/potassium-transporting ATPase subunit alpha-2 | 5.08E-11 |
|  | Gene_Symbol=ATP1B1 Isoform 1 of Sodium/potassium-transporting ATPase subunit beta-1 | 0.000 |
|  | Gene_Symbol=ATP5A1 ATP synthase subunit alpha, mitochondrial | 0.000 |
| Transferase | Gene_Symbol=GALNT6 Polypeptide N-acetylgalactosaminyltransferase 6 | 0.000 |
|  | Gene_Symbol=RPN1 Dolichyl-diphosphooligosaccharide--protein glycosyltransferase subunit 1 precursor | 0.000 |
|  | Gene_Symbol=B3GNT2 Isoform 2 of UDP-GlcNAc:betaGal beta-1,3-N-acetylglucosaminyltransferase 2 | 0.000 |
|  | Gene_Symbol=RPN2 Dolichyl-diphosphooligosaccharide--protein glycosyltransferase subunit 2 | 0.000 |
|  | Gene_Symbol=FUT8 Isoform 1 of Alpha-(1,6)-fucosyltransferase | 0.000 |
|  | Gene_Symbol=B4GALT5 Beta-1,4-galactosyltransferase 5 | 0.000 |
| Receptor | Gene_Symbol=M6PR Cation-dependent mannose-6-phosphate receptor | 0.000 |
|  | Gene_Symbol=GRB10 Isoform 3 of Growth factor receptor-bound protein 10 | 0.000 |
|  | Gene_Symbol=CNP Isoform CNPI of 2',3'-cyclic-nucleotide 3'-phosphodiesterase | 0.000 |
|  | Gene_Symbol=PLD3 Phospholipase D3 | 0.000 |
|  | Gene_Symbol=HS2ST1 Isoform 2 of Heparan sulfate 2-O-sulfotransferase 1 | 0.000 |
| Transporter | Gene_Symbol=SLC3A2;LOC442497 Isoform 2 of 4F2 cell-surface antigen heavy chain | 0.000 |
|  | Gene_Symbol=SLC1A5 Neutral amino acid transporter B(0) | 0.000 |
|  | Gene_Symbol=SLC2A1 Solute carrier family 2, facilitated glucose transporter member 1 | 0.000 |
| Integrin | Gene_Symbol=ITGA6 Isoform Alpha-6X1X2B of Integrin alpha-6 | 0.000 |
| Adhension | Gene_Symbol=ICAM1 Intercellular adhesion molecule 1 | 0.000 |
| Cadherin | Gene_Symbol=CDH4 Cadherin-4 | 0.000 |
|  | Gene_Symbol=- 14 kDa protein | 0.000 |
|  | Gene_Symbol=BSG Isoform 2 of Basigin | 0.000 |
|  | Gene_Symbol=TMEM87A Isoform 2 of Transmembrane protein 87A | 0.000 |
|  | Gene_Symbol=JUP Junction plakoglobin | 0.000 |
|  | Gene_Symbol=MFI2 Uncharacterized protein | 0.000 |
|  | Gene_Symbol=LMNA Isoform A of Prelamin-A/C | 0.000 |
|  | Gene_Symbol=HSPA5 HSPA5 protein | 0.000 |
|  | Gene_Symbol=KRT9 Keratin, type I cytoskeletal 9 | 0.000 |
|  | Gene_Symbol=ADAM9 Isoform 1 of Disintegrin and metalloproteinase domain-containing protein 9 | 0.000 |
|  | Gene_Symbol=CD46 Isoform B of Membrane cofactor protein | 0.000 |
|  | Gene_Symbol=RDX Radixin, isoform CRA_a | 0.000 |
|  | Gene_Symbol=MSN Moesin | 0.000 |
|  | Gene_Symbol=MFI2 Uncharacterized protein | 0.000 |
|  | Gene_Symbol=TMPO Isoform Beta of Lamina-associated polypeptide 2, isoforms beta/gamma | 0.000 |
|  | Gene_Symbol=CANX cDNA FLJ55574, highly similar to Calnexin | 0.000 |
|  | Gene_Symbol=TGOLN2 Isoform TGN51 of Trans-Golgi network integral membrane protein 2 | 0.000 |
|  | Gene_Symbol=NCLN Isoform 1 of Nicalin | 0.000 |
|  | Gene_Symbol=UQCRC2 Cytochrome b-c1 complex subunit 2, mitochondrial | 0.000 |
|  | Gene_Symbol=STOML2 Stomatin-like protein 2 | 0.000 |
|  | Gene_Symbol=ZMPSTE24 CAAX prenyl protease 1 homolog | 0.000 |
|  | Gene_Symbol=TM9SF2 Transmembrane 9 superfamily member 2 | 0.000 |

By immunoprecipitation, the antigens were captured by mAb 201E4 from AGS cell lysate. Then, the antigens were identified by mass spectra assay.
